# Altered Functional Connectivity of Fusiform Gyrus in Subjects with Amnestic Mild Cognitive Impairment: A Resting-State fMRI Study

**DOI:** 10.3389/fnhum.2015.00471

**Published:** 2015-08-27

**Authors:** Suping Cai, Tao Chong, Yun Zhang, Jun Li, Karen M. von Deneen, Junchan Ren, Minghao Dong, Liyu Huang, for the Alzheimer’s Disease Neuroimaging Initiative

**Affiliations:** ^1^School of Life Science and Technology, Xidian University, Xi’an, China

**Keywords:** fusiform gyrus, face recognition, visual cognition, amnestic mild cognitive impairment, functional connectivity, functional MRI

## Abstract

Visual cognition such as face recognition requests a high degree of functional integration between distributed brain areas of a network. It has been reported that the fusiform gyrus (FG) is an important brain area involved in facial cognition; altered connectivity of FG to some other regions may lead to a deficit in visual cognition especially face recognition. However, whether functional connectivity between the FG and other brain areas changes remains unclear in the resting state in amnestic mild cognitive impairment (aMCI) subjects. Here, we employed a resting-state functional MRI (fMRI) to examine alterations in functional connectivity of left/right FG comparing aMCI patients with age-matched control subjects. Forty-eight aMCI and 38 control subjects from the Alzheimer’s disease Neuroimaging Initiative were analyzed. We concentrated on the correlation between low frequency fMRI time courses in the FG and those in all other brain regions. Relative to the control group, we found some discrepant regions in the aMCI group which presented increased or decreased connectivity with the left/right FG including the left precuneus, left lingual gyrus, right thalamus, supramarginal gyrus, left supplementary motor area, left inferior temporal gyrus, and left parahippocampus. More importantly, we also obtained that both left and right FG have increased functional connections with the left middle occipital gyrus (MOG) and right anterior cingulate gyrus (ACC) in aMCI patients. That was not a coincidence and might imply that the MOG and ACC also play a critical role in visual cognition, especially face recognition. These findings in a large part supported our hypothesis and provided a new insight in understanding the important subtype of MCI.

## Introduction

Mild cognitive impairment (MCI), often a progressive state between normal aging and Alzheimer’s disease (AD), is a higher at-risk state for AD (Petersen et al., 2001). Recent studies showed that individuals with MCI tended to develop to AD at a rate of about 10–15% per year (Misra et al., 2009), compared with the normal aged people who develop AD at a rate of 1–2% per year (Bischkopf et al., 2002). The subtypes of MCI have been proposed several years ago based on the models of neuropsychological impairment (Winblad et al., 2004). Amnestic mild cognitive impairment (aMCI) is one of the MCI subtypes which is ­characterized by primary memory deficits and has a high ­progression to AD (Petersen, 2004; Petersen et al., 2014). In recent years, the subtype of aMCI in particular has been obtained much attention (Fleisher et al., 2007; Whitwell et al., 2008; Zhang et al., 2015).

Visual cognition deficits were reported to accompany the development of AD (Kiyosawa et al., 1990; Mendez et al., 1990a; Cronin-Golomb, 1995). Visual symptoms include impaired facial recognition (Sedwick, 1987), impaired discrimination or recognition of familiar objects (Kaskie and Storandt, 1995), and impaired visuo-spatial abilities (Mendez et al., 1990b). Mangun et al. (1998) have demonstrated that the function of visual cognition has a relationship with the FG, and dysfunction in the FG leads to obstruction of visual cognition, which have been confirmed by previous studies (Butter et al., 1996; Golby et al., 2005). Bokde et al. (2006) reported that MCI patients had widespread changes in fusiform connectivity during the performance of a face-matching task. In a combined resting-state functional and structural MRI study, He et al. (2007) found altered low-frequency (0.01 Hz < *f* < 0.08 Hz) blood oxygenation level-dependent fluctuations coherence in the FG in the AD patients. Besides, previous research observed that both AD and MCI patients had functional activation changes in the FG during a visual working memory task relative to the normal controls (Yetkin et al., 2006). In addition, considering the structural aspect, several studies have demonstrated that neurofibrillary tangles (NFTs) have a strong relationship to dementia severity in AD (Price et al., 1991; Giannakopoulos et al., 1995). Guillozet et al. (2003) found that there were clusters of tangles in the FG, and MCI subjects showed higher NFTs densities than those in normal age-matched control subjects. As pathological changes worsen, NFTs become more numerous in this region. Recent studies have demonstrated that structural changes could potentially effect on functional disorders in patients (He et al., 2007; Oakes et al., 2007), so that higher NFT densities may lead to abnormal function in the FG. These findings confirmed that fusiform lesions contributed to AD disease such as in the visual cognition disorder mentioned above. However, one fact is that this symptom is not only related to the dysfunction in the FG but also to some other brain regions which may have a connection with the FG. In other words, a specific cognitive function, such as visual cognition requests a high degree of functional integration between distributed brain areas to support this cognitive function. It reminded us to use a popular method to explore our idea, and this method is called functional connectivity which represents the synchronized neural activity between brain regions (Biswal et al., 1995) and has been widely used in the brain imaging community to study the functional integration of the brain (Wang et al., 2013; Cai et al., 2014).

Here, we intended to use the FG as the seed region and make a correlation analysis (Pearson correlation) between it and other regions in the whole brain. We explored whether the functional connectivity of the left/right fusiform gyri to the rest of the brain regions showed differences between the aMCI and control subjects. Moreover, we noticed that almost all of these studies were carried out under the task-state. However, in the resting state, little attention has been devoted to functional connectivity of the fusiform gyri in aMCI patients. Therefore, we attempted to supplement this kind study. We hypothesized functional connectivity of the FG would be altered in aMCI patients.

## Materials

### Overview of ADNI

Data used in the preparation of this article were obtained from the Alzheimer’s disease Neuroimaging Initiative (ADNI) database (adni.loni.usc.edu). ADNI was launched in 2003 by the National Institute on Aging (NIA), the National Institute of Biomedical Imaging and Bioengineering (NIBIB), the Food and Drug Administration (FDA), and private pharmaceutical companies and non-profit organizations, as a $60 million, 5-year public–private partnership. The primary goal of ADNI has been to test whether serial magnetic resonance imaging, positron emission tomography (PET), other biological markers, and clinical and neuropsychological assessment can be combined to measure the progression of MCI and early AD. For up-to-date information, see www.adni-info.org.

### Subjects

This study was carried out in accordance with the recommendations of ADNI database with written informed consent from all subjects. All 86 subjects (48 aMCI and 38 control subjects) from the ADNI cohort were formally evaluated using eligibility criteria. The ADNI database obtained the written informed consent from all subjects or their surrogates. The information regarding ethics approval for the study is in the Supplementary Material. Briefly, experienced clinicians conducted independent semi-structured interviews with the participant and a knowledgeable collateral source that included a health history, neurological examination, the mini-mental state examination (MMSE) (Cockrell and Folstein, 2002), and the CDRSum of Boxes (Morris, 1993). Subjects from the ADNI database were selected if they were clinically classified as: (a) control subjects, individuals who were cognitively normal at baseline and clinical follow-up (CDR 0); (b) aMCI, individuals with mild cognitive impairment defined using the revised MCI criteria (Petersen, 2004) and showed primary memory deficits on neuropsychological examinations. Data from nine subjects (four control subjects, five aMCI) were removed owing to head-motion problem (see Data Preprocessing). The details of clinical and demographic information for the remaining 77 (43 aMCI, 34 control subjects) participants are shown in Table 1.

**Table 1:** **Demographics and clinical information**.

	aMCI (*n* = 43)	CG (*n* = 34)	*P* value
Gender (male/female)	19/24	15/19	1^a^
Age (years)	72.35 ± 8.78	75.63 ± 5.70	0.228^b^
MMSE score	24.70 ± 1.97	28.65 ± 2.01	<0.01^b^
CDR score	0.51 ± 0.13	0.00 ± 0.00	<0.01^c^

*Values are means ± SD*.

*^a^value was obtained by a two-tail Pearson chi-square test*.

*^b^value was obtained by a two-sample t-test*.

*^c^value was obtained by a one-sample -test*.

*MMSE, mini-mental state examination; CDR, rate of clinical dementia; aMCI, amnesic mild cognitive impairment; CG, control group*.

### fMRI data and T1 data acquisition

The raw Digital Imaging and Communications in Medicine (DICOM) resting-state functional images that we used were downloaded from the ADNI database^1^, which were scanned on a 3.0-T MRI scanner. The data were acquired by using an EPI (echo-planar imaging sequence) (Delapaz, 1994), with data parameters as follows: 140 time points; TR is 3000 ms; TE is 30 ms; flip angle (FA) is 80°, 48 slices; slice thickness = 3.3 mm, spatial resolution = 3 mm×3 mm×3 mm and matrix = 64×64. The data information is similar with one of our pervious study (Cai et al., 2014). T1-weighted images were acquired using a sagittal magnetization prepared rapid gradient echo (MP-RAGE), with data parameters as follows: slice thickness is 1.2 mm, TR is 6700 ms, TE is 3.1 ms, FA is 9°, matrix = 256×256×170. For more information about the data, see http://adni.loni.usc.edu/.

## Methods

### Data preprocessing

Prior to the statistical analyses, data preprocessing was performed using a MATLAB toolbox called DPARSF (Data Processing Assistant for Resting-State fMRI)^2^ (Chao-Gan and Yu-Feng, 2010), which was based on some functions in SPM8 (Statistical Parametric Mapping)^3^ and REST (Resting-State fMRI Data Analysis Toolkit)^4^ (Song et al., 2011). For each subject, we discarded the first 10 time points to avoid transient signal changes before magnetization reached steady-state and subjects’ adaptation to the functional MRI (fMRI) scanning noise. The rest images were conducted slice-timing correction for different signal acquisition, and then realigned to the first volume to correct for head motion. Subjects with head motion exceeding 1.5 mm in any dimension of *x*, *y*, and *z* or 1.5° in any angular motion were excluded for further analysis. Next, we spatially normalized images to Montreal neurological institute (MNI) space by individual T1 anatomical images which had been registered to the mean functional image and re-sampled them to a voxel size of 3 mm×3 mm×3 mm. Then, the functional images were spatially smoothed with a Gaussian kernel of 4 mm full width at half maximum (FWHW) to decrease spatial noise. Subsequently, we removed the linear trends to wipe out any residual effects of motion and other non-neuronal factors, and applied a temporally filter (0.01 Hz < *f* < 0.08 Hz) to the time series of each voxel to decrease the impact of high-frequency noise (Cordes et al., 2001; Greicius et al., 2003). Subjects with head motion exceeding 1.5 mm in any dimension or 1.5° in any angular motion through the resting-state run were excluded for further analysis, hence a total of nine subjects (five aMCI, four control subjects) were excluded. Finally, a Friston-24 parameter^5^ (Friston et al., 1996; Yan et al., 2013), as well as parameters for the global mean signal, cerebrospinal fluid signal, and white matter signal, was regressed as nuisance covariates for following analysis.

Recently, it has been reported that micro head movement may have impact on certain resting-state fMRI metrics, such as the method of functional connectivity (Power et al., 2012; Satterthwaite et al., 2012; Van Dijk et al., 2012; Yan et al., 2013). Therefore, we, respectively, evaluated the correlation between the time series of the left/right fusiform and frame-wise displacement (FD) according to the standard of Power et al. (2012) to exam the influence of the head motion on the functional connectivity analysis. Then, we converted the correlation coefficients to *z* values using Fourier’s transform to ameliorate the normality. Finally, we estimated the between-group differences in head motion.

### Using amplitude of the low-frequency fluctuation analysis to define seed regions

**Figure 1:**
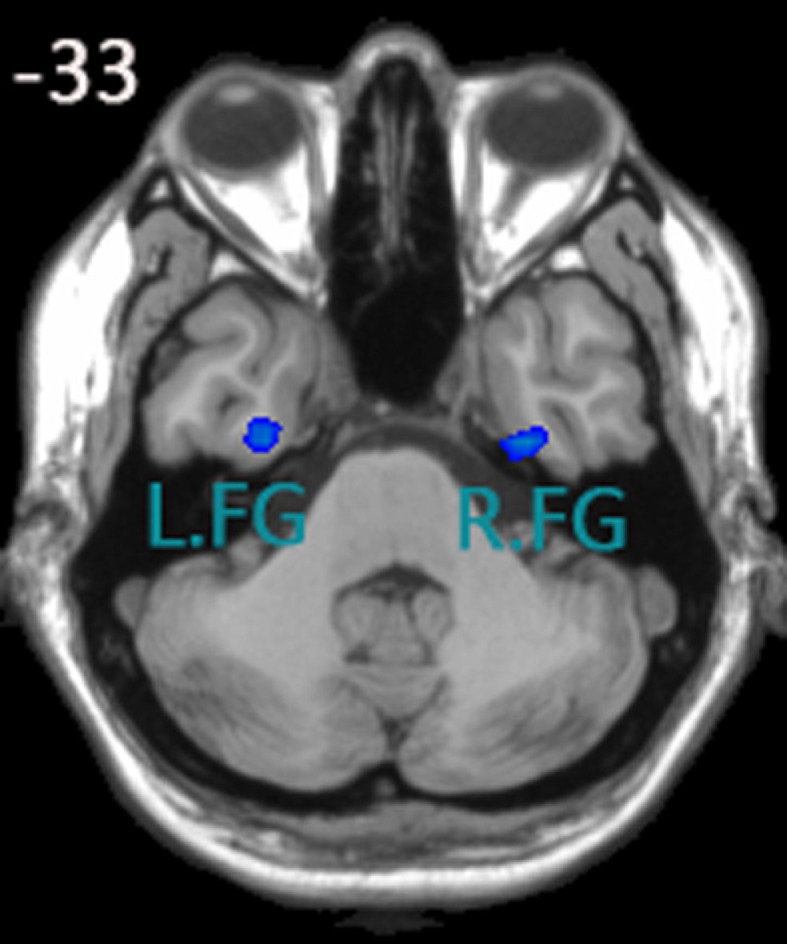
**Seed regions of the left FG and right FG;** (1) x, y, z coordinates of primary peak locations in the space of MNI of the left FG are −28, −9, −38; t = −2.731; volume: 1836 mm3. (2) x, y, z coordinates of primary peak locations in the space of MNI of the right FG are 32, −10, −38; t = −2.935;volume: 2403 mm3.

Yu-Feng et al. (2007) reported that amplitude of low-frequency (0.01–0.08 Hz) fluctuation (ALFF) is able to reflect the baseline brain function and could be used as a biomarker to assess spontaneous brain activity. Several studies employed it to measure the alterations in baseline brain activity for both healthy subjects (Zuo et al., 2010) and subjects with pathological conditions (Liu et al., 2012). Thus, we conducted the ALFF analysis using REST software to define the seed regions. The calculation procedure was similar as Yu-Feng et al. (2007)’s study. Subsequently, a voxel-wise two-sample *t*-test was carried out to compare the mALFF differences between the control group and aMCI group using REST 1.8 software. As was expected, one of the brain regions showing a significant difference between ALFF values was the FG (*P* < 0.05, false discovery rate corrected). We chose the left and right fusiform gyri as our seed regions (left FG volume: 1836 mm^3^, right FG volume: 2403 mm^3^), for more details, see Figure 1. Each seed region was re-sampled to obtain a new seed region image with the same spatial resolution as the preprocessed fMRI images using the 0 interpolation approach. Following this step, we carried out binarized transformation for each seed region to refrain the non-zero value.

### Functional connectivity analysis

In the two groups, the blood oxygen level dependent (BOLD) time courses of the seed regions were averaged to produce the reference time courses. A Pearson correlation analysis was conducted between the reference time series and those of all of the other brain regions. Then, we converted the correlation coefficients to *z* values using Fourier’s transform to ameliorate the normality (Lowe et al., 1998).

### Within-group and between-group statistical analysis

The *z* value of each subject was conducted a one-sample *t*-test in a voxel-wise mode. In the two groups, a single voxel threshold value of *P* < 0.01 and a minimum cluster size of 60 contiguous voxels was used to correct for multiple comparisons. This yielded a corrected threshold of *P* < 0.01 in the two groups, as determined by the Monte Carlo simulation (see program AlphaSim by D. Ward in AFNI software. Parameters were as follows: single voxel *P* value = 0.01, FWHM = 4 mm, with mask).

Subsequently, we restricted our analyses to the survived regions of a one-sample *t*-test from the above within-group analyses. We also conducted statistical comparisons of the functional connectivity values between the two groups using a two-sample *t*-test and with age as the covariance. The statistical maps of the two groups were corrected by the Monte Carlo simulation resulting in a corrected threshold of *P* < 0.01.

### Detailed conditions of altered functional connectivity about identified areas

In the current study, the altered functional connectivity includes the decreased and increased functional connectivity. In more detail, “Decreased functional connectivity” could be due to (1) decrease in negative functional connectivity (increase in absolute value); (2) turn from positive functional connectivity into negative functional connectivity; and (3) decrease in positive functional connectivity (decrease in absolute value); The similar situation applies to “increased functional connectivity.” To present the condition of the brain areas we identified in detail, we extracted the correlation coefficients. Then, we did the statistical analysis by using an SPSS one-sample *t*-test.

### Relationship between functional connectivity values and clinical data

We extracted the connectivity strength from the identified regions that had differences between the two groups (see Tables 3 and 4). Correlation analyses between these functional connectivity strengths and MMSE were conducted to estimate whether the functional connectivity values had a relationship with clinical data.

## Results

Table 1 shows the demographic characteristics. There were no significant differences in gender and age, but the MMSE score was significantly different (*P* < 0.01) between the two groups and CDR score was significantly greater than zero (*P* < 0.01) in the aMCI subjects. There was no correlation between time courses of the left/right FG and FD in each group. We also estimated the between-group differences in micro head motion. The results showed that there were no significant differences [*P* = 0.169 (left FG); *P* = 0.624 (right FG), obtained by a two-sample two-tailed *t*-test]. For more details, see Table 2.

**Table 2:** **The correlation between frame-wise displacement (FD) and time series of the left/right fusiform gyrus in the two groups**.

	aMCI_L	aMCI_R	CG_L	CG_R	aMCI-CG_L	aMCI-CG_R
*r* (m ± SD)	0.0083 ± 0.0548	−0.0084 ± 0.0564	0.0078 ± 0.0706	0.0096 ± 0.0576		
*P*	0.325^a^	0.332^a^	0.523^c^	0.339^a^	0.169^b^	0.624^b^
*t*	0.997	−0.982	−0.645	−0.97	1.389	0.492

*The correlation coefficient *r* is given as mean ± SD (m ± SD)*.

*CG, control group; aMCI_L/R, the correlation between FD and time series of the left/right fusiform gyrus in aMCI; CG_L/R, the correlation between FD and time series of the left/right fusiform gyrus in control group; aMCI-CG_L/R, the differences between aMCI and the control group in head motion*.

*^a^value was obtained by a one-sample -test*.

*^b^value was obtained by a two-sample -test*.

### Within-group functional connectivity analyses

**Figure 2:**
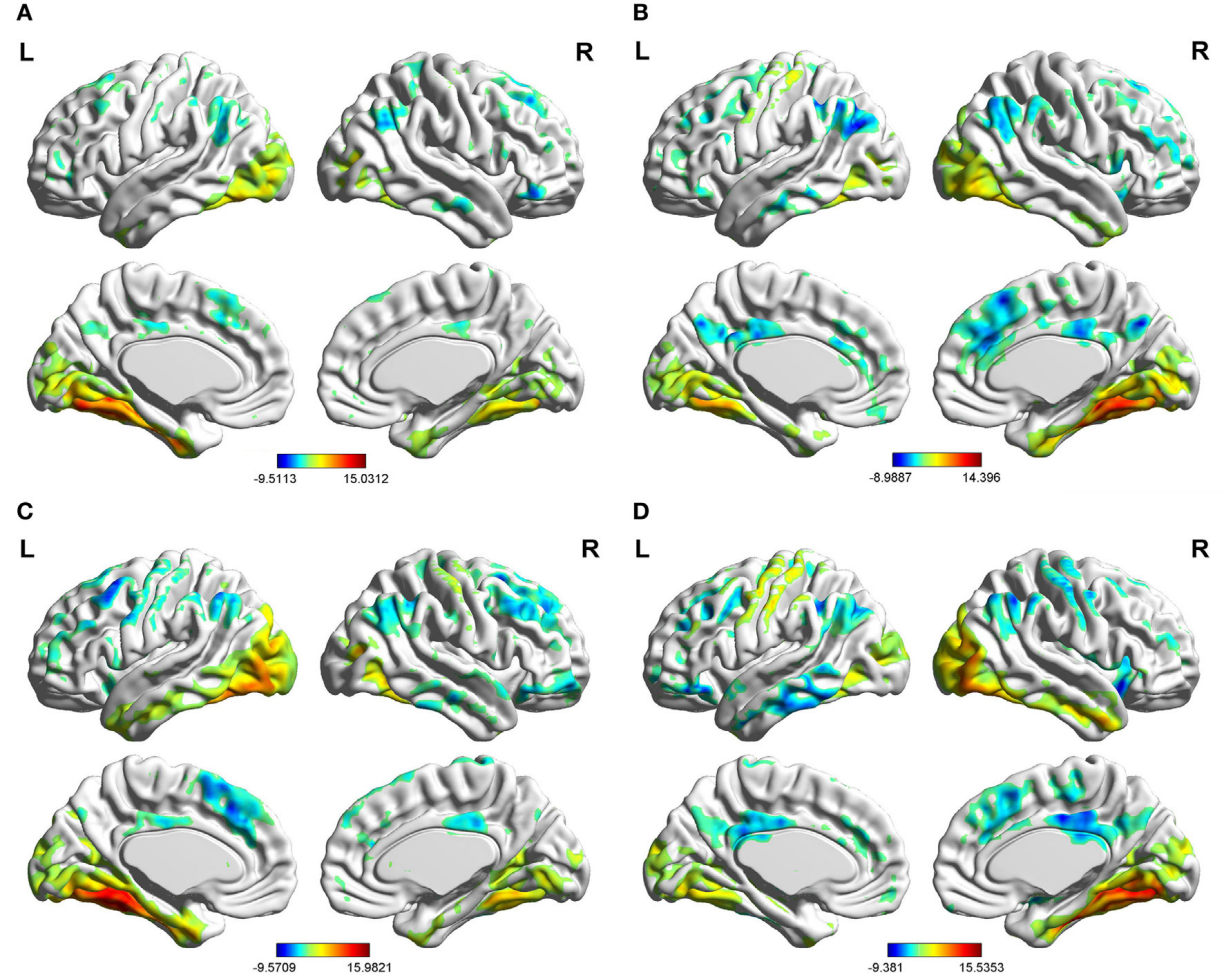
**Brain areas showing significant connectivity to the left and right fusiform gyri in the aMCI group and control group (*P* **<** 0.01, 60 voxels, Alphasim corrected). (A)** control group in the left fusiform gyrus; **(B)** control group in the right fusiform gyrus; **(C)** aMCI group in the left fusiform gyrus; **(D)** aMCI group in the right fusiform gyrus.

In the two groups, we found significant functional connectivity between the left/right FG and in between them (Figure 2). In the age-matched normal control group, the left (Figure 2A) and right (Figure 2B) fusiform gyri showed significant connectivity to a set of regions. Among these regions, the middle occipital gyrus (MOG), lingual gyrus (LING), inferior temporal gyrus (ITG), and calcarine cortex are located in the visual perception network (Allen et al., 2011). The medial prefrontal cortex (MPFC), anterior cingulate cortex (ACC), posterior cingulate cortex (PCC), precuneus (PreCU), ITG, and inferior parietal lobule (IPL) overlap with regions underlying the default mode network (DMN) (Greicius et al., 2003). Visual inspection of the connectivity maps revealed that the magnitude and extent of right FG connectivity to other brain regions were both greater than those of the left FG in the control group. All the results that we obtained applied a one-sample, two-tailed *t*-test in a voxel-wise manner and combined threshold clusters with 60 voxels at a corrected level of *P* < 0.01. For more details, see Tables S1–S4 in Supplementary Material and Figure 2.

### Between-group functional connectivity analyses

The last three columns of Table 3 show the detailed condition about the altered connectivity for the regions that we identified. For example, the *t* value between the left PreCU and left FG in the aMCI group was −0.3910, but in the control group it was −4.3530. We obtained that the left PreCU had increased negative functional connectivity (decrease in absolute value) when comparing aMCI with the control group. Similar explanations are the same with the other identified brain areas.

**Table 3:** **Regions showing functional connectivity differences of the left/right fusiform gyrus between the aMCI and control group (*P* **<** 0.01, 28 voxels, corrected for multiple comparisons)**.

Brain regions	BA	Cluster size	MNI			Max T2	CG	aMCI	
			*x*	*y*	*z*		*T1*	*T1*	
**FUNCTIONAL CONNECTIVITY OF THE LEFT FUSIFORM GYRUS**									
**aMCI < CG**									
R.FG (+)	37	61	33	−75	−15	−3.8803	7.891	3.195	↓+
**aMCI > CG**									
L.MOG (+)	19	134	−27	−90	24	3.6043	1.606	6.852	↑+
L.LING (+)	19	93	−29	−61	2	3.8262	2.581	5.697	↑+
L.PreCU (−)	7	41	−9	−60	39	3.1048	−4.353	−0.391	↑−
R.ACC (−)	33	22	6	21	18	3.6612	−2.675	−1.28	↑−
R.THAL (+)	–	56	−8	−16	17	3.6910	2.364	5.031	↑+

**aMCI < CG**	**BA**	**Cluster size**	***x***	***y***	***z***	**Max *T2***	***T1***	***T1***	

**FUNCTIONAL CONNECTIVITY OF THE RIGHT FUSIFORM GYRUS**									
L.ITG (+)	37	65	−48	−42	−18	−3.6456	6.439	1.166	↓+
L.FG (+)	37	42	23	−51	−13	−3.2262	9.954	8.258	↓+
L.LING (+)	17	74	3	−73	6	−4.1071	5.804	2.717	↓+
L.ParaHip (+)	34	28	−12	−6	−24	−4.0105	3.121	0.336	↓+
**aMCI > CG**									
R.SMG (+)	2	42	65	−22	29	3.858	1.684	4.325	↑+
L.SMA (+)	32	51	−5	11	47	4.03	1.923	6.471	↑+
L.MOG (+)	19	94	−39	−84	30	2.9629	3.955	5.336	↑+
R.ACC (−)	24	33	9	30	9	4.5676	−3.588	−0.506	↑−

*CG, control group; FG, fusiform gyrus; MOG, middle occipital gyrus; LING, lingual gyrus; PreCU, precuneus; ACC, anterior cingulate cortex; THAL, thalamus; ITG, inferior temporal gyrus; ParaHip, parahippocampus; SMG, supramarginal gyrus; SMA, supplementary motor area; L, left; R, right; BA, Brodmann’s area; x, y, z coordinates of primary peak locations in the space of MNI; “+”: positive functional connectivity; “−”: negative functional connectivity; **T2** value was obtained by a two-tailed two-sample t-test. The last three columns reveal the detailed condition of decreased and increased connectivity for the identified regions; **T1** value was obtained by a one-sample t-test; “↓+”, decrease in positive functional connectivity; “↑+”, increase in positive connectivity; “↑−”, increase in negative connectivity*.

**Figure 3:**
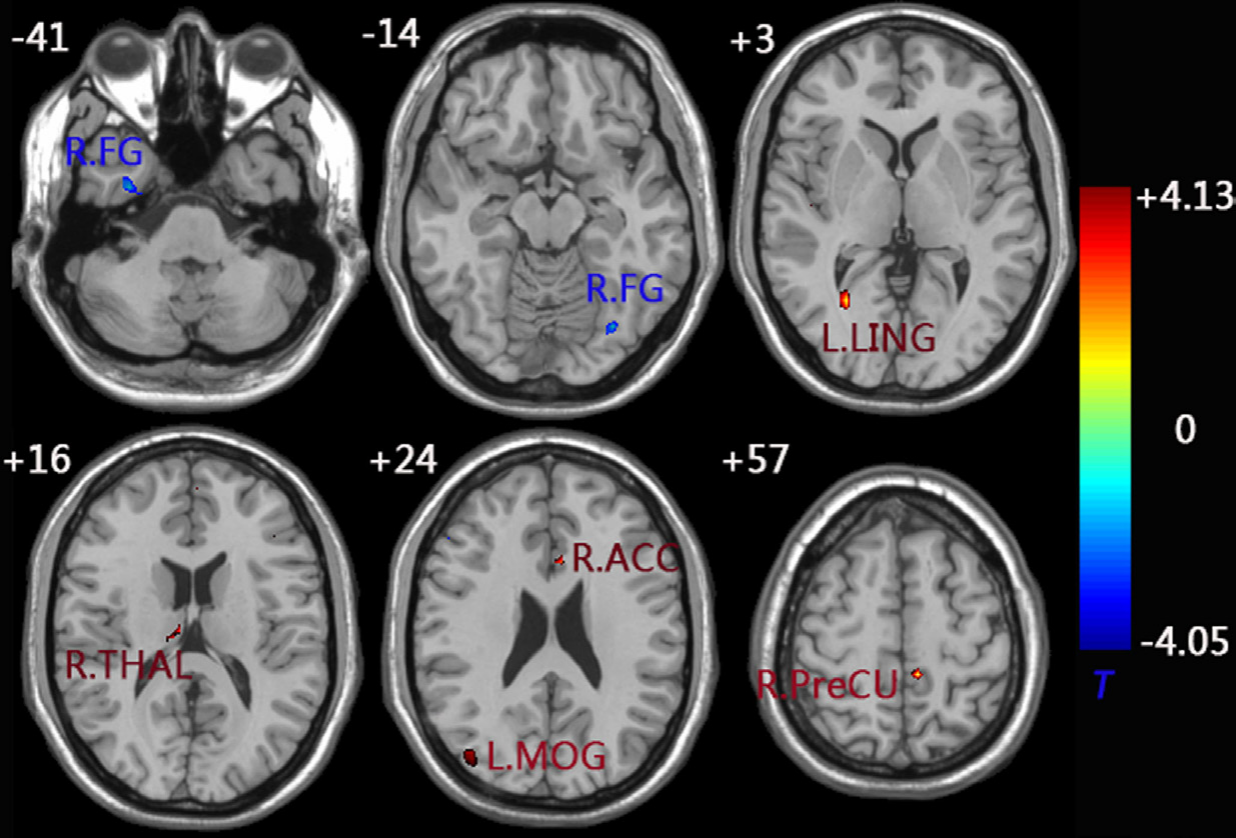
**functional connectivity difference maps of the left FG between the aMCI and control group; (*P* < 0.01, 28 voxels, corrected for multiple comparisons)**. Abbreviations: FG, fusiform gyrus; MOG, middle occipital gyrus; LING, lingual gyrus; PreCU, precuneus; ACC, anterior cingulate cortex; THAL, thalamus; L, left; R, right.

When compared to the control group, the aMCI group demonstrated decreased functional connectivity between the left FG and right FG. In comparison, increased functional connectivity was observed between the left FG and a group of brain regions including the left MOG, left LING, left PreCU, right ACC, and right thalamus (THAL) (Figure 3 and Table 3).

**Figure 4:**
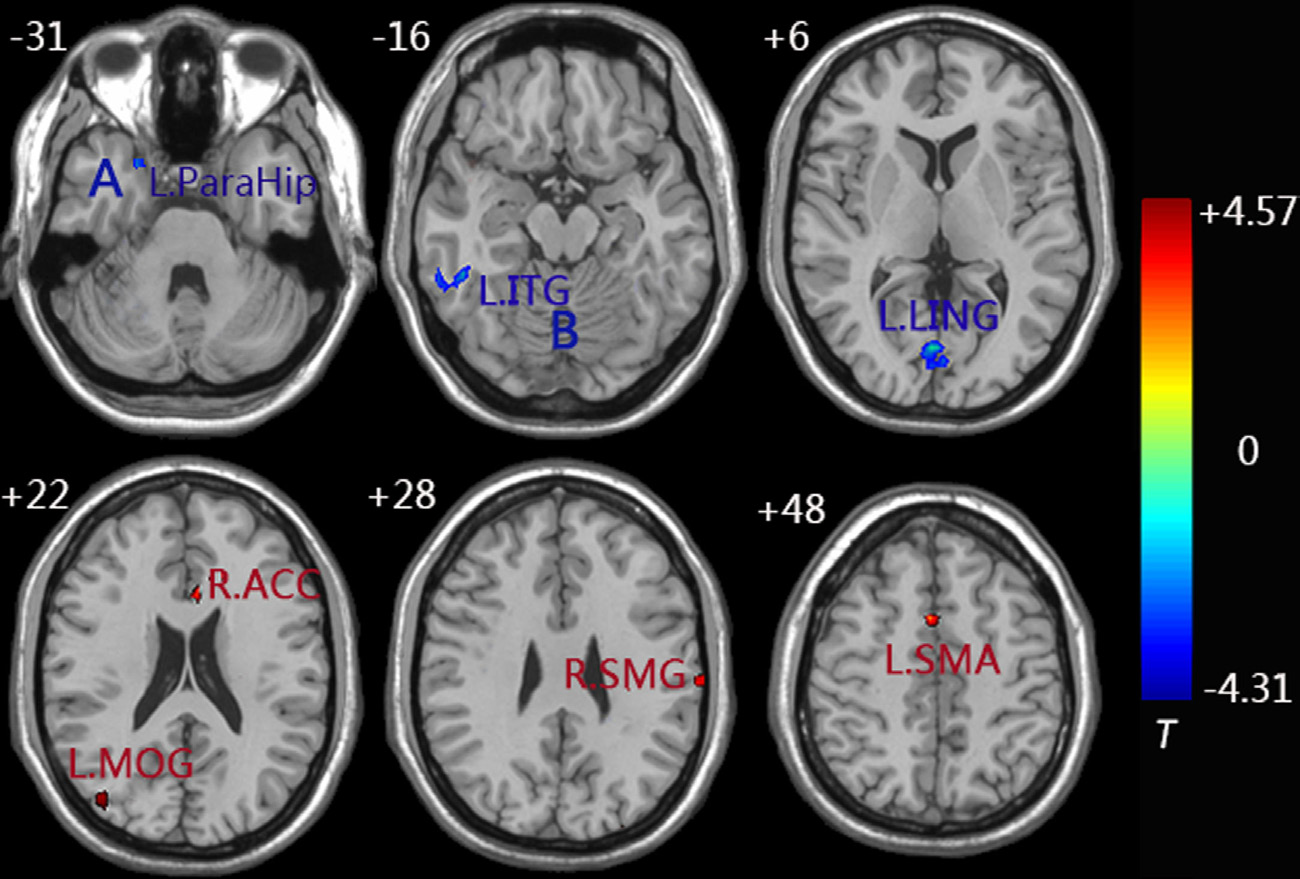
**Functional connectivity difference maps of the right FG between the aMCI and control group; (*P* < 0.01, 28 voxels, corrected for multiple comparisons);** Abbreviations: MOG, middle occipital gyrus; LING, lingual gyrus; ACC, anterior cingulate cortex; ITG, inferior temporal gyrus; ParaHip, parahippocampus; SMG, supramarginal gyrus; SMA, supplementary motor area; L, left; R, right; Letter A represents the left ParaHip and letter B represents the left ITG.

Similarly, decreased functional connectivity between the right FG and brain regions was detected in the regions of the left FG, left ITG, left parahippocampus (ParaHip), and left LING. Increased functional connectivity between the right FG and brain regions were found in the regions of the right supramarginal gyrus (SMG), left supplementary motor area (SMA), left MOG, and right ACC. More importantly, we noticed that both the left MOG and right ACC had an increased connection with the left/right FG compared to the control group (Figure 4 and Table 3).

### Relationship between functional connectivity and clinical variables

**Figure 5:**
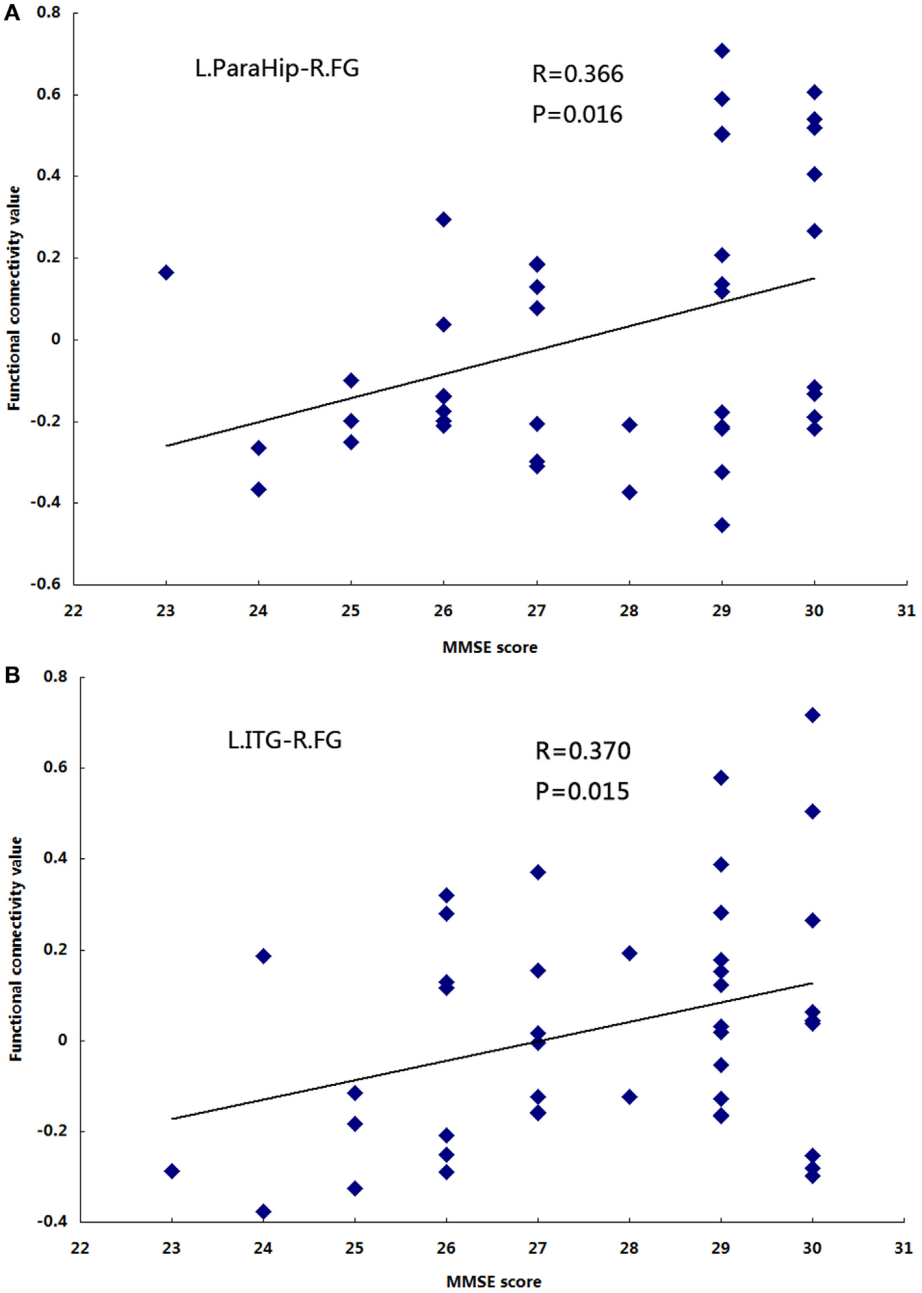
**The correlation between the functional connectivity values and MMSE scores in the aMCI group (*P* **<** 0.05)**. **(A)** L.ParaHip-R.FG: the strength of functional connectivity between the left ParaHip and right FG; **(B)** L.ITG-R.FG: the strength of functional connectivity between the left ITG and right FG. For details, please see Table 4.

Significant positive correlations were observed between functional connectivity values in the left ParaHip (*r* = 0.366; *P* = 0.016) and left ITG (*r* = 0.370; *P* = 0.015), and MMSE scores. No significant correlations between MMSE scores and functional connectivity values were found in the other brain regions we identified. For more information, see Table 4 and Figure 5.

**Table 4:** **This table shows the correlation coefficient and corresponding *P* value between the functional connectivity values and MMSE scores; the results for a threshold of *P* **<** 0.05 are shown in bold**.

	ROIs	Brain area	aMCI	
			*CC*	*P*
MMSE	L.FG	L.LING	0.201	0.086
		R.THAL	0.124	0.112
		R.FG	0.066	0.674
		L.MOG	0.065	0.68
		L.PreCU	0.131	0.842
		R.ACC	0.095	0.487
	R.FG	**L.ITG**	**0.370**	**0.015**
		L.FG	0.103	0.513
		L.LING	0.052	0.742
		**L.ParaHip**	**0.366**	**0.016**
		R.SMG	0.069	0.786
		L.SMA	0.135	0.324
		L.MOG	0.116	0.458
		R.ACC	0.036	0.819

*FG, fusiform gyrus; MOG, middle occipital gyrus; LING, lingual gyrus; PreCU, precuneus; ACC, anterior cingulate cortex; THAL, thalamus; ITG, inferior temporal gyrus; HIP, hippocampus; ParaHip, parahippocampus; SMG, supramarginal gyrus; SMA, supplementary motor area; L, left; R, right; CC: correlation coefficient*.

*The P value was obtained by a one-sample t-test*.

## Discussion

The objective of our study was to explore the features of the left/right FG functional connectivity with the rest of brain regions in aMCI and age-matched control group by measuring resting-state fMRI signals. Compared to the control group, we found some discrepant regions in the aMCI group which presented increased or decreased connectivity with the left/right FG including ITG, LING, ParaHip, FG, MOG, ACC, PreCU, SMG, SMA, and THAL. Moreover, the discrepant brain regions were primarily involved in visual cognition. More importantly, we also obtained that both left and right FG have increased functional connections with the left MOG and right ACC in the aMCI group. That was not a coincidence and might imply that the MOG and ACC also play a critical role in visual cognition. These findings in a large part supported our hypothesis and provided a new insight on the important subtype of aMCI.

### Functional connectivity pattern of the fusiform gyrus in aMCI and control group

Note that functional connectivity of the left/right FG includes positive and negative connectivity, and the connectivity pattern of these two groups is different in terms of both intensity and quantity. Several studies have demonstrated that negative functional connectivity may represent an anti-correlation effect, which is suppressed during activation of the FG, or the anti-correlation effect intrinsically exists between the two opposed networks (Murphy et al., 2009; Stevens et al., 2010). On the contrary, the positive functional connectivity may represent that these regions exist in the same network. Nevertheless, the correct interpretation of positive or negative functional connectivity is still controversial. We will explore this problem in the future.

Compared to the control group, we also found functional connectivity strength between the FG and some other brain regions presented decreased and increased in aMCI. The next section will discuss our findings in more detail.

### Decreased functional connectivity of the fusiform gyrus in aMCI

In the current study, we detected decreased functional connectivity between fusiform and ITG. Structurally, the ITG is located in the end of the ventral visual stream (Tanaka, 1996) and this region is deemed to be the storage site of long-term visual memory (Miyashita, 1993). Moreover, we learned that the FG are also located in ventral visual stream (Puce et al., 1995). Thomas et al. (2009) used diffusion tensor imaging (DTI) and tractography and detected a tract that through the core fusiform region to the anterior temporal gyrus. Face recognition is integrated in temporal cortical areas, especially in the FG and anterior temporal gyrus (Nasr and Tootell, 2012). A recent study combined fMRI and repetitive trans-cranial magnetic stimulation in control group and observed convergent evidence for a pivotal role of the FG and ITG in a visual semantic task (Binney et al., 2010). Convit et al. (2000) suggested that the ITG could be the first temporal regions affected in AD and volume shrinkage in this area could predict the occurrence of future AD among non-demented persons. In addition, the functional connectivity strength between the right FG and ITG was positively correlated with MMSE scores (Table 4 and Figure 2B), which manifested that cognitive competence have a notable relationship with the functional connectivity value between two brain regions. In other words, the positive correlation indicates that a lower MMSE value corresponding to a lower functional connectivity value, a lower MMSE value means more serious disease, and a lower functional connectivity value signifies a functional deficit in functional imaging. Interestingly, the changed functional connectivity between the ITG and FG is also consistent with Yao et al. (2010)’s study where altered interregional correlations were detected in the FG and ITG. Likewise, we also detected decreased functional connectivity between the LING and FG. The LING is also an important brain region to process visual function and bilateral destruction of the LING can affect complex visual processing. However, deficit of the LING alone is not sufficient to lead to impaired visual processing. A study reported that the FG has a critical role in visual processing, such as facial recognition and corresponding to visual imagery (Mangun et al., 1998), and together the function of the FG with the LING would be complete visual cognition. Impaired visual cognition in aMCI patients may be due to decreased connectivity between the FG and ITG/LING, which we observed in our study. This viewpoint supported and extended several researchers’ studies showing that AD can impact many aspects of visual processing, such as the face recognition and perceptual discrimination, colors, orientation, spatial frequency, and objects (Cronin-Golomb et al., 1993; Kurylo et al., 1994; Rizzo et al., 2000).

Similarly, the right FG has shown decreased functional connectivity with left ParaHip, which is the principal regions of neuronal degeneration in Alzheimer’s disease (Braak et al., 2006). In more detail, the neuropathological biomarkers of AD, NFTs and neuritic plaques first appeared in the entorhinal cortex (Braak and Braak, 1996; Simic et al., 1997) and then extend to the ParaHip and other neocortical regions. Moreover, according to previous research using a structural imaging (Fox and Schott, 2004), functional imaging (Buckner et al., 2000; Rombouts et al., 2000; Sperling et al., 2003), post-mortem examination (Braak and Braak, 1996), and resting metabolism (Herholz, 2003), explicit memory deficits in AD have relationship with functional and structural abnormalities of the ParaHip. FG is located at the end of the ventral visual stream (Puce et al., 1995) which is also a higher-order visual cortex. Golby et al. (2005) demonstrated that there were prominent deficits in a higher-order FG and ParaHip and activation in these two regions were correlated with explicit memory performance. In addition, Mion et al. (2010) observed that the degree of semantic deficit was related to dysfunction of the FG/ParaHip. This finding is also consistent with post-mortem examination studies which estimated correlation between semantic scores and volume atrophy (Murayama et al., 2010). More importantly, we found that the functional connectivity values between the right FG and left ParaHip were positively correlated with MMSE scores (Table 4; Figure 2A). The convergence of our results and these previous reports suggested that abnormal functional connectivity between the FG and ParaHip was contributed to the deficit of explicit memory and semantic memory in aMCI patients.

We also observed decreased functional connectivity between the right FG and the left FG in the aMCI group compared to that in the control group. Several studies reported that AD/MCI group had significant atrophy of the FG relative to controls using voxel-based morphometry (Chételat et al., 2002, 2005; Bozzali et al., 2006). Recently, several studies have demonstrated that structural changes could potentially effect on functional disorders in patients (He et al., 2007; Oakes et al., 2007). Thus, the decreased functional connectivity between the right FG and the left FG might be due to the structural atrophy of the FG. According to MTI news (January 9, 2012), the left and right FG have different roles in cognition, and they are mutually connected. The left FG is responsible for identifying characteristics of a visual object which are similar to the face and the right is responsible for determining whether the similar face is a human face or a familiar face. Therefore, the abnormal connectivity between the two sides of the FG might be due to the interactive information barriers of the bilateral FG. Furthermore, the decreased connectivity between them could explain the symptom that AD/aMCI patients cannot recognize familiar faces. What is more, our results were partly consistent with Bokde et al. (2006)’s study showing that there is decreased connectivity between the right middle FG and left middle FG in the MCI group compared to that in the healthy control group during a face-matching task.

### Increased functional connectivity of the fusiform gyrus in aMCI

In this study, both the MOG and ACC have a positive linear correlation with the left/right FG, indicating that MOG, ACC, and FG are in the same network. The ACC sits in a unique position in the prefrontal lobe and likely has a significant role in integration of neuronal circuitry for affect regulation (Eslinger et al., 2004). The MOG is sited in the primary visual cortex and appeared to be involved in processing visual recognition. This finding was not a coincidence and might imply that the MOG and ACC also play a critical role in visual cognition. A result from a previous study combined DTI and fMRI could support this finding: Sala-Llonch et al. (2015) explored functional and structural connectivity of visuo-perceptual working memory and found that axial diffusivity and fractional anisotropy of the tracts connecting the fusiform gyri with the prefrontal areas extending to part of the ACC and middle occipital cortex. However, in the aMCI group, we observed increased connections between the left/right FG and right ACC. Previous studies have shown that the decreased volume of ACC occurring in aMCI patients and AD group had significant atrophy of the FG relative to controls using volumetric analysis (Chételat et al., 2002; Bozzali et al., 2006). Hafkemeijer et al. (2013) demonstrated that increased functional connectivity has relationship with brain atrophy in elderly with subjective memory complaints. Thus, the increased connectivity between FG and ACC might be attributed to brain atrophy of the two regions. Other than the increased connections between the FG and ACC, we also found increased connectivity between the FG and MOG. Haxby et al. (2000) demonstrated a central system including the occipital face area and the fusiform face area in the ventral occipital-temporal cortex which responds preferentially to faces over other visual objects. Using DTI and tractography, a previous study found that a breakdown in structural connectivity in ventral occipital-temporal cortex and suggested that the abnormal structural connectivity may be the structural basis for the deficit in facial recognition (Thomas et al., 2009). The disruptive structural connectivity might be the pathological structure foundation of abnormal functional connectivity (increased connectivity) (Greicius et al., 2009). In addition, we could explain the increased connectivity between FG and MOG from another angle. Golby et al. (2005) reported that the AD group presented deficient explicit memory but had intact implicit memory, and the intact implicit encoding in the occipital cortex. The increased connectivity between MOG and FG might help to guarantee intact implicit memory in patients. Of course, this is just a conjecture, so we will provide more accurate research in the future.

The PreCU has a negative correlation with the left FG. According to previous studies, the PreCU is a part of the DMN. Connectivity of the DMN plays a key role in the human cognition (Greicius et al., 2003). In the current study, we obtained increased connectivity between the FG and PreCU in the aMCI group relative to control group. Karas et al. (2007) detected the PreCU atrophy in early state of AD using a morphometric structural MRI method. Bozzali et al. (2006) also obtained the FG had significant atrophy in MCI group relative to controls using volumetric analysis. The increased functional connectivity has relationship with brain atrophy in elderly with subjective memory complaints which has been demonstrated by Hafkemeijer et al. (2013). Thus, the increased connectivity between FG and PreCU might be due to volume atrophy of the two areas. Recent research used graph theory and resting-state fMRI methods to study the topology structure of the functional connectivity in AD or/and MCI, and observed that decreased and increased brain regions were mainly located in the DMN (Zhao et al., 2012; Wang et al., 2013). As in our study, the increased resting-state FG connectivity with PreCU is consistent with these previous findings and is also in accord with Grady et al. (2003)’s assumption that AD/MCI patients could recruit the other resources to form a reimbursement mechanism.

In addition, we also found the fusiform has connection with the SMG and SMA in the two groups. Using a DTI tractography approach, Haas et al. (2012) demonstrated that the FG has wide connectivity with a set of brain areas such as the visual processing areas, the frontal, and parietal cortex. SMG is a main sub-division of the inferior parietal cortex which plays a great part in visual word recognition (Rushworth et al., 2006). Stoeckel et al. (2009) reported that the SMG is functionally and specifically involved in retrieval of visual word memory. Interestingly, the same research (Stoeckel et al., 2009) also found that SMG was not an isolated region activated when subjects executed visual tasks and activations were also found in the occipital-temporal regions such as the FG, post-central areas, and SMA. In other words, the FG, SMG, and SMA are in the same network. Moreover, Grignon et al. (1998) detected cytoarchitectonic alterations in the SMG of late onset AD. Therefore, changes in the structure of SMG may spread to changes in functional connection with the other regions in the same network (He et al., 2007; Oakes et al., 2007). We also detected increased connectivity between the right SMA and right FG. This finding suggested that the abnormal functional connectivity was spread to the supplementary motor cortex owing to this disease.

We also obtained the left FG has increased functional connectivity with the right THAL. According to a previous study, the THAL is a pivotal site that integrates various neural activities from widespread cortical inputs and outputs and is considered to modulate communication with distributed brain regions (Postuma and Dagher, 2006). Previous studies based on brain imaging demonstrated significant connectivity between the THAL and different brain regions, such as the frontal, temporal, parietal, and occipital lobes (Behrens et al., 2003; Johansen-Berg et al., 2005; Zhang et al., 2008). Some of these connections, such as the thalami-fusiform connection, are considered to be important pathways for visual memory processing. In aMCI patients, the THAL initiates its regulating function and has enhanced connectivity with the left FG. This evidence of increased connectivity between the two regions has also been interpreted as a recruitment of additional neural resources (recruitment of the right THAL) to compensate for losses of cognitive functions.

### Limitations

There are several limitations in the current study. First, several studies have demonstrated that structural changes could potentially effect on functional disorders in patients (He et al., 2007; Oakes et al., 2007). In the future, we will use our own experimental data by combining functional connectivity with structural connectivity techniques to explore the correlativity between the two aspects. Second, in the current study, we extracted connectivity strength from the identified regions that have differences between the two groups and performed correlation analyses between functional connectivity strength and MMSE (Cockrell and Folstein, 2002). The MMSE consists of the following seven aspects: time, direction of site orientation, immediate memory, attention and computing capacity, delay memory, language, and visual and spatial ability. Actually, we should make correlation analyses between functional connectivity strength and scores of visual ability. However, the public database only provides the total points of the MMSE, and scores of sub-item categories are not made public. We will use our own experimental clinical data to explore the correlation between functional connectivity strength and scores of visual ability in our future study. Finally, we removed the global signal as this regressor might be associated variance in fMRI functional connectivity analyses (Desjardins et al., 2001). However, there is much debate about whether regressing the global mean signal. We will explore this issue in the future.

## Conclusion

In summary, our present work studied the FG functional connectivity with all the other brain regions between aMCI and an age-matched control group during the resting state. We found that there was remarkable differentia in the functional connectivity of FG between the two groups. Moreover, the discrepant brain regions were primary involved in visual cognition. More importantly, compared to the control group connections with the left middle occipital gyrus (MOG) and right anterior cingulate cortex (ACC) in the aMCI group. That was not a coincidence and might imply that the MOG and ACC also play a critical role in visual cognition. These findings in some ways supported our hypothesis and provided a new sight in understanding the important subtype of aMCI.

## Conflict of Interest Statement

The authors declare that the research was conducted in the absence of any commercial or financial relationships that could be construed as a potential conflict of interest.

## Supplementary Material

The Supplementary Material for this article can be found online at http://journal.frontiersin.org/article/10.3389/fnhum.2015.00471

## Footnotes

^1^http://adni.loni.usc.edu/

^2^http://www.restfmri.net

^3^http://www.fil.ion.ucl.ac.uk/spm

^4^http://www.restfmri.net

^5^Head movement is a confounding factor for functional connectivity studies. Although previous research includes six head motion parameters as nuisance variables to remove the effect of the motion, many recent studies suggested trying to control for head movement except for basic head-movement regression, see Power et al. (2012); Satterthwaite et al. (2012); Van Dijk et al. (2012); Yan et al. (2013) for discussion of this problem and possible corrections. Yan et al. (2013) suggested that individual-level correction with the Friston-24 model is recommended.

## Abbreviations

ACC, anterior cingulate cortex; ALFF, amplitude of the low-frequency fluctuation; aMCI, amnestic mild cognitive impairment; FG, fusiform gyrus; ITG, inferior temporal gyrus; LING, lingual gyrus; MOG, middle occipital gyrus; ParaHip, parahippocampus; PreCU, precuneus; THAL, thalamus; SMA, supplementary motor area; SMG, supramarginal gyrus.
