# Supplementary material for: Altered Functional Connectivity of Fusiform Gyrus in Subjects with Amnestic Mild Cognitive Impairment: A Resting-State fMRI Study
Source: Front Hum Neurosci. 2015 Aug 27;9:471. doi: 10.3389/fnhum.2015.00471 (PMC4550786; doi:10.3389/fnhum.2015.00471)
Supplement: Supplementary file 1 [file data_sheet_1.doc]

**Supplementary materials:**

1. **The information regarding ethics approval for the study:**

(1) This study was carried out in accordance with the recommendations of Alzheimer’s disease Neuroimaging Initiative (ADNI) database (adni.loni.usc.edu) with written informed consent from all subjects. All 86 subjects (48 MCI and 38 control subjects) from the ADNI cohort were formally evaluated using eligibility criteria. Written informed consent was obtained from all participants or surrogates.

(2) We complied with their requirement to “submit all manuscripts to the ADNI Data and Publications Committee (DPC) prior to submitting to a journal”. Following are our email communication.

*On Dec. 12, 2014, at 8:10 AM, Suping Cai wrote:*

*Dear Erin Drake,*

*Thanks for the ADNI sharing the data. I submit all manuscripts to the ADNI Data and Publications Committee (DPC) prior to submitting to a journal for the review.*

*I download data from the ADNI database for the purposes of analysis. On the by-line of the manuscript, after the named authors, I included the phrase “for the Alzheimer’s Disease Neuroimaging Initiative”, include language in the methods section in order to accurately acknowledge data gathering by the ADNI personnel and acknowledge funding by the ADNI in the support acknowledgement section of the manuscript. The detailed article is in the attachment.*

*I look forward to hearing from you. If there are any questions, please contact with me.*

*Kind regards.*

*Suping Cai*

*ADNI Data wrote back：*

*Dear Cai,*

*Thank you! On behalf of the Alzheimer's Disease Neuroimaging Initiative Data and Publications Committee (ADNI DPC), I would like to thank you for sending your manuscript in for review and for following the ADNI Publications Policy so precisely. Your paper was reviewed and is acceptable for submission to a journal.*

*Please send a copy of your manuscript by email to me after it has been accepted for publication. At that time, you should also provide a copy of your published manuscript to the National Institute on Aging by emailing it to Ms. Margaret Vaughn at vaughnms@mail.nih.gov.*

*Again, thank you for submitting your manuscript to the ADNI DPC. We wish you luck with your submission!*

*Thanks,*

*Erin Drake*

*edrake@genetics.med.harvard.edu*

Table S1

Location of statistically significant functional connectivity peaks of the left FG in aMCI group (*P* < 0.01, 60 voxels, corrected for multiple comparisons)

|  |  |  | MNI | | |  |
| --- | --- | --- | --- | --- | --- | --- |
| Brain regions | BA | Cluster size | x | y | z | Max ***t*** |
| PALL | -- | 49 | -9 | -6 | -9 | 4.5618 |
| R.FG | 37 | 120 | 23 | -51 | -13 | 14.0312 |
| L.MOG | 19 | 110 | -33 | -51 | -18 | 15.9821 |
| L.SPL | 7 | 52 | -24 | -57 | 54 | 4.8295 |
| R.ACC | 24 | 46 | 12 | 0 | 9 | -6.8908 |
| R.THA | -- | 60 | 12 | 0 | 9 | -6.8902 |
| L.IPL | 40 | 168 | -54 | -42 | 51 | -9.5709 |
| L.PreCU | 7 | 57 | -3 | -72 | 42 | -4.8945 |
| Cerebelum_8 | -- | 51 | 15 | -36 | -63 | -5.149 |
|  | -- | 33 | 12 | -60 | -39 | -5.0232 |

Abbreviations: PALL: pallidum; FG: fusiform gyrus; SPL: superior parietal lobe; ACC: anterior cingulum cortex; THA: thalamus; IPL: inferior parietal lobe; PreCU: Precuneus; L: left; R: right;

Table S2

Location of statistically significant functional connectivity peaks of the left FG in control group (*P* < 0.01, 60 voxels, corrected for multiple comparisons)

|  |  |  | MNI | | |  |
| --- | --- | --- | --- | --- | --- | --- |
| Brain regions | BA | Cluster size | x | y | z | Max ***t*** |
| PALL | -- | 49 | -9 | -12 | -9 | 4.7186 |
| R.ParaHip | 36 | 47 | 24 | -3 | -33 | 5.7851 |
| R.FG | -- | 61 | -36 | -36 | -21 | 15.9821 |
| R.INS | -- | 35 | 36 | 15 | 12 | 4.5551 |
| R.MPFC | 6 | 181 | 12 | 30 | 54 | -9.5113 |
| R.CAU | -- | 108 | 18 | 15 | 9 | -7.1144 |
| L.PreCU | 7 | 133 | -3 | -72 | 42 | -4.9652 |
| R.MFG | -- | 95 | 42 | 45 | 21 | -5.359 |
| L.ANG | 39 | 124 | -45 | -57 | 24 | -6.7969 |
| R.ANG | 40 | 172 | 54 | -54 | 33 | -7.9011 |
| R.STG | -- | 65 | 48 | -12 | -3 | -6.1384 |
| R.MTG | -- | 72 | 66 | -12 | -24 | -6.3557 |
| R.ACC | 6 | 91 | 12 | 30 | 54 | -9.5113 |
| R.PoCG | -- | 62 | 21 | -36 | 72 | -5.6586 |

Abbreviations: PALL: pallidum; ParaHip: parahippocampal; FG: fusiform gyrus; INS: Insula; MPFC: medial prefrontal lobe; CAU: caudate nucleus; PreCU: Precuneus; MFG: middle frontal gyrus; ANG: angular gyrus; STG: superior temporal gyrus; MTG: middle temporal gyrus; ACC: anterior cingulum cortex; PoCG: postcentral gyrus; L: left; R: right.

Table S3

Location of statistically significant functional connectivity peaks of the right FG in aMCI group (*P* < 0.01, 60 voxels, corrected for multiple comparisons)

|  |  |  | MNI | | |  |
| --- | --- | --- | --- | --- | --- | --- |
| Brain regions | BA | Cluster size | x | y | z | Max ***t*** |
| R.THA | -- | 53 | 15 | -27 | 12 | 7.3332 |
| R.PALL | -- | 36 | 12 | 3 | -6 | 4.8351 |
| L.FG | 37 | 126 | 36 | -21 | -30 | 8.258 |
| R.FG | -- | 218 | -31 | -43 | -12 | 13.41 |
| L.ITG | 19 | 80 | -25 | -54 | -5 | 5.779 |
| L.MOG | -- | 81 | -35 | -81 | 11 | 6.262 |
| R.MOG | -- | 137 | 40 | -80 | 17 | 8.697 |
| R.HIP | -- | 98 | -35 | -23 | -14 | 5.16 |
| L.ParaHip | 36 | 106 | 24 | -34 | -13 | 5.876 |
| R.MFG | -- | 196 | 48 | 24 | 45 | -9.381 |
| R.INS | 13 | 177 | 30 | 18 | -9 | -8.9455 |
| L.THA | -- | 121 | -6 | -12 | -18 | -7.2221 |

Abbreviations: THA: thalamus; PALL: Pallidum; FG: fusiform gyrus; ITG: inferior temporal gyrus;

MOG: middle occipital gyrus; HIP: hippocampus; ParaHip: parahippocampal; MFG: middle frontal gyrus; INS: Insula; L: left; R: right;

Table S4

Location of statistically significant functional connectivity peaks of the right FG in control groups (*P* < 0.01, 60 voxels, corrected for multiple comparisons)

|  |  |  | MNI | | |  |
| --- | --- | --- | --- | --- | --- | --- |
| Brain regions | BA | Cluster size | x | y | z | Max ***t*** |
| R.THA | -- | 47 | 15 | -24 | 15 | 4.167 |
| L.FG | 37 | 126 | -37 | -58 | -17 | 9.954 |
| R.FG | 37 | 219 | -32 | -43 | -12 | 12.33 |
| L.MOG | 19 | 104 | 39 | -81 | -1 | 5.668 |
| L.ITG | 20 | 129 | -30 | -36 | -17 | 10.78 |
| R.LING | 19 | 89 | 19 | -52 | -1 | 10.03 |
| L.LING | 18 | 76 | -15 | -73 | -1 | 7.526 |
| L.CAL | 17 | 67 | 7 | -82 | 13 | 5.297 |
| R.CAL | 18 | 90 | -10 | -89 | 13 | 5.761 |
| R.HIP | -- | 52 | -31 | -34 | -8 | 6.782 |
| L.ParaHip | 37 | 49 | 27 | -38 | -10 | 7.646 |
| L.CAU | -- | 158 | -18 | 18 | 9 | -7.473 |
| R.PCC | 31 | 78 | -5 | -52 | 30 | -4.889 |

Abbreviations: THA: thalamus; FG: fusiform gyrus; MOG: middle occipital gyrus; ITG: inferior temporal gyrus; LING: lingual gyrus; CAL: calcarine cortex; HIP: hippocampus; ParaHip: parahippocampal; CAU: caudate nucleus; PCC: posterior cingulate cortex; L: left; R: right;
